# Prognostic impact of molecular muscle-invasive bladder cancer subtyping approaches and correlations with variant histology in a population-based mono-institutional cystectomy cohort

**DOI:** 10.1007/s00345-021-03788-1

**Published:** 2021-07-14

**Authors:** Veronika Weyerer, Robert Stoehr, Simone Bertz, Fabienne Lange, Carol I. Geppert, Sven Wach, Helge Taubert, Danijel Sikic, Bernd Wullich, Arndt Hartmann, Markus Eckstein

**Affiliations:** 1 Institute of Pathology, University Hospital Erlangen, Friedrich-Alexander Universität Erlangen-Nürnberg, Krankenhausstr. 8-10, 91054 Erlangen, Germany; 2 Comprehensive Cancer Center Erlangen (CCC ER-EMN), University Hospital Erlangen, Friedrich-Alexander-Universität Erlangen-Nürnberg, Erlangen, Germany; 3 Department of Urology and Pediatric Urology, University Hospital Erlangen, Friedrich-Alexander Universität Erlangen-Nürnberg, Erlangen, Germany

**Keywords:** Muscle-invasive bladder cancer, Molecular subtypes, Prognostic impact, Histological variants

## Abstract

**Purpose:**

Recently discovered molecular classifications for urothelial bladder cancer appeared to be promising prognostic and predictive biomarkers. The present study was conducted to evaluate the prognostic impact of molecular subtypes assessed by two different methodologies (gene and protein expression), to compare these two approaches and to correlate molecular with histological subtypes in a consecutively collected, mono-institutional muscle-invasive bladder cancer (MIBC) cohort.

**Methods:**

193 MIBC were pathologically re-evaluated and molecular subtypes were assessed on mRNA (NanoString technology, modified 21-gene-containing MDACC approach) and protein levels (immuno-histochemical [IHC] analysis of CK5, CK14, CD44, CK20, GATA3 and FOXA1). Descriptive statistical methods and uni-/multi-variable survival models were employed to analyze derived data.

**Results:**

Neither gene expression nor protein-based subtyping showed significant associations with disease-specific (DSS) or recurrence-free survival (RFS). Agreement between mRNA (reference) and protein-based subtyping amounted 68.6% for basal, 76.1% for luminal and 50.0% for double-negative tumors. Histological subtypes associated with RFS in uni-variable (*P* = 0.03), but not in multivariable survival analyses. Tumors with variant histology predominantly showed luminal subtypes (gene expression subtyping: 36/55 cases, 65.5%; protein subtyping: 44/55 cases, 80.0%). Squamous differentiation significantly associated with basal subtypes (gene expression subtyping: 44/45 squamous cases, 97.8%; protein subtyping: 36/45 cases, 80.0%).

**Conclusion:**

In our consecutive cystectomy cohort, neither gene, protein expression-based subtyping, nor histological subtypes associated with DSS or RFS in multi-variably adjusted survival analyses. Application of a limited IHC subtyping marker panel showed high concordance of 83.9% with gene expression-based subtyping, thus underlining the utility for subtyping in pathological routine diagnostics. In addition, histological MIBC subtypes are strong indicators for intrinsic subtypes.

**Supplementary Information:**

The online version contains supplementary material available at 10.1007/s00345-021-03788-1.

## Introduction

Urothelial bladder cancer (UBC) is among the tenth-most common cancers worldwide, and is a prime example of carcinogen-derived cancer with smoking representing the main risk factor [1]. Based on the depth of invasion, UBC is histologically classified as non-(NMIBC) as well as muscle-invasive bladder cancer (MIBC) [2]. Additionally, these histologically defined borders associate with distinct molecular and clinical behaviors: 80% of bladder tumors are NMIBC, frequently recur and often harbor *FGFR3* alterations as oncogenic drivers. In contrast, MIBC is a life-threatening disease with five-year survival rates between 40 and 60% and is mainly driven by *TP53* mutations [3].

Modern high-throughput genomic technologies rapidly led to new insights in molecular heterogeneity of UBC, e.g., distinct luminal, basal and neuroendocrine molecular subtypes identified by transcriptional analyses. Several widely overlapping subtyping approaches with heterogeneous nomenclatures have been proposed mainly for MIBC [4–9], which were summarized into six consensus subtypes in 2020 coordinated by the Bladder Cancer Molecular Taxonomy Group (BCMTG) [10]. The discovery of these three major molecular phenotypes, luminal, basal and neuroendocrine-like tumors, represents a milestone in bladder cancer research. Luminal tumors can be subdivided into less aggressive “papillary”—like tumors exhibiting expression profiles of non-invasive high-grade UBC, along with two further subtypes, which share an EMT gene signature and are highly invasive [8, 10]. Basal tumors exhibit gene expression related to squamous differentiation and are highly proliferative while neuroendocrine carcinomas show classical expression of neuroendocrine differentiation markers [8, 10]. While earlier studies suggested an improved response to neoadjuvant chemotherapy (NAC) in basal carcinomas [11], more recent findings indicate that NAC response is rather dependent on immune infiltration than intrinsic differentiation [12]. However, until today, validation of the utility of different subtyping approaches (mRNA vs. protein) in consecutive population-based cohorts is still rare and heavily needed to explore its true predictive and prognostic potential. Importantly, smaller case series also suggested that subtypes correlate with histological subtypes/variant histologies of MIBC [2, 5, 6, 8, 10, 13, 14], but systematic analysis in coherent cohorts is lacking.

Here, we studied the prognostic impact of two subtyping approaches, i) a gene expression-based classifier (adapted MDACC approach [5]), and ii) a protein-based classifier (consensus panel by the BCMTG) in a consecutive population-based mono-institutional cohort of 193 patients treated with radical cystectomy and bilateral lymphadenectomy. Furthermore, subtype assignments were correlated with histological MIBC subtypes.

## Materials and methods

### Study population

We studied 193 MIBC cases of the Comprehensive Cancer Center Erlangen Metropole Region Nuremberg (CCC-EMN) cohort collected from 2004 until 2016. As previously, described, included patients were treated with radical cystectomy and bilateral lymph node dissection in curative intent [15–17]. 56 patients received adjuvant platinum-based chemotherapy within 3 months after cystectomy when patients recovered from surgery. No patient was treated with neoadjuvant chemotherapy. All cases were routinely mapped for approximately 23 standardized positions as described previously [18, 19]. All cases were systematically reviewed by two experienced uropathologists (A.H., M.E.) according to the latest TNM staging manual of the UICC 8th edition, 2017 and the WHO 2016 classification for tumors of the genitourinary tract [2]. Histological variants were reported if at least 10% of tumor showed variant histology including pure and mixed tumors [20, 21]. Clinico-pathologic characteristics of the analyzed cohort are shown in Supplementary Table S1. Ethical approval for this study was obtained by the ethical review board of the Friedrich-Alexander-University Erlangen-Nürnberg (Erlangen, Germany; approval number: no. 3755 and 329_16B). All patients gave informed consent, and all analyses were carried out in accordance with the Declaration of Helsinki.

### RNA isolation and gene expression-based molecular classification

FFPE sections with at least 50% tumor content were used for isolation. As previously described, RNA was isolated with Maxwell Promega RNA purification kits (Promega). The mRNA expression of target genes was determined via nCounter^®^ MAX/FLEX system (NanoString Technologies^®^, USA). To differentiate luminal, basal and double-negative phenotypes, a customized 21-gene-containing nCounter^®^ PlexSet^™^ (NanoString Technologies^®^) according to the MD Anderson Cancer Center (MDA) subtyping approach was applied as described previously [5, 17]. Selected genes are summarized in Supplementary Table 2. Gene counts were normalized using two reference genes (*SDHA*, *HPRT1*) and log2-transformed for further analysis using the nSolver 4.0 software (NanoString).

### Immuno-histochemical analysis (IHC) and protein-based molecular classification

IHC was performed using 4 µm tissue microarray sections, which were previously reported and contain punches covering the invasion front as well as the tumor center, on a Ventana BenchMark Ultra (Ventana, USA) and a Dako Link 48 (Dako, USA) auto-stainer in immunohistochemistry laboratories with accreditation by the German Accreditation Office (DAKKs) according to DIN EN ISO/IEC 17020 [15, 17]. To identify luminal and basal subtypes, we applied a six-marker panel consisting of CK5 (clone XM26, mouse monoclonal, Diagnostic BioSystems^®^, USA, dilution 1:50), CK14 (clone SP53, rabbit monoclonal, Cell Marque^™^, USA, dilution 1:40), CK20 (clone Ks 20.8, mouse monoclonal, Dako, Denmark, dilution 1:50), GATA3 (clone L50-823, mouse monoclonal, DCS, Germany, dilution 1:100), FOXA1 (rabbit polyclonal ab23738, Abcam^®^, United Kingdom, dilution 1:400), and CD44 (clone DF1485, mouse monoclonal, Dako, dilution 1:50) based on the recommendations provided by the BCMTG [22]. Stained slides were scanned on a slide scanner (P250 Flash, 3DHistech, Hungary) and tumor areas on TMA cores were annotated by two pathologists (ME, VW) in QuPath (https://qupath.github.io). TMA cores were quality-controlled for presence of tumor tissue and proper staining results. Cores with either absence of representative tumor tissue or staining artifacts (e.g., DAB shades) were excluded from the analysis. Expression of subtyping markers was automatically detected as H-score (scale 0–300) via QuPath. For further analysis, median H-scores were log2-transformed.

### Statistical analysis

Cluster analysis was performed by unsupervised hierarchical clustering based on Ward clustering method using Euclidean distance as metric scale. Disease-specific (DSS) and recurrence-free survival (RFS) curves were estimated by the Kaplan–Meier method. Log-rank test was used for time-to-event outcomes. Multivariable survival analyses were conducted using Cox proportional hazards regression modeling to assess the magnitude of impact while adjusting for well-known clinico-pathological risk parameters (gender, age, resection margin, WHO 1973 Grading, WHO 2016 Grading, lymphovascular invasion, blood vessel invasion, perineural sheath invasion, receipt of adjuvant platinum-based chemotherapy, variant histology). All *P* values were two-sided, and a *P* value < 0.05 was considered statistically significant. All statistical analyses were performed by GraphPad Prism 8.3.0 (GraphPad Software Inc., La Jolla, CA, USA) and JMP SAS 13.2 (SAS, Cary, NC, USA).

## Results

### Gene expression-based molecular analysis and associations with clinical outcomes

**Fig. 1:**
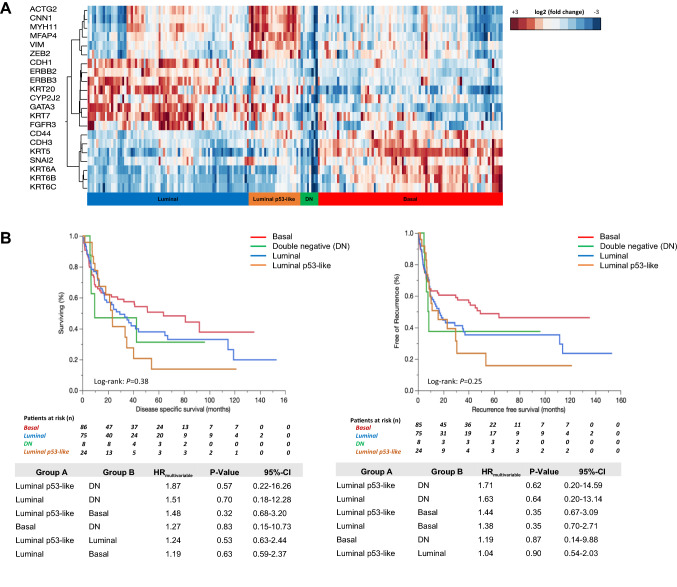
**A** Unsupervised hierarchical cluster analysis reveals four different mRNA-based subtyping cluster: luminal, luminal p53-like, basal, double-negative (DN) were identified. **B** Univariable Kaplan–Meier regressions of disease-specific (DSS) and recurrence-free survival (RFS) based on gene expression derived subtypes. Table shows total patients/phenotype with the following columns showing the number of patients at risk in 20-month increments. Multivariable Hazard risk (HR) ratios are shown

Unsupervised hierarchical clustering of the 21 target genes (adapted MDACC subtyping panel) revealed four distinct cluster groups: 86 tumors were assigned as basal (44.6%), 75 as luminal (38.9%), 24 as luminal p53-/ECM-like (12.4%) and 8 as double-negative (DN; 4.1%; Fig. 1A). Assignments to these classes did not correlate significantly with DSS or RFS neither in univariable Kaplan–Meier regressions (DSS: log-rank *P* = 0.38; RFS: log-rank *P* = 0.25) nor in multivariable Cox-regression analyses (Fig. 1B).

### IHC-based molecular subtypes in MIBC and their associations with classification approaches

**Fig. 2:**
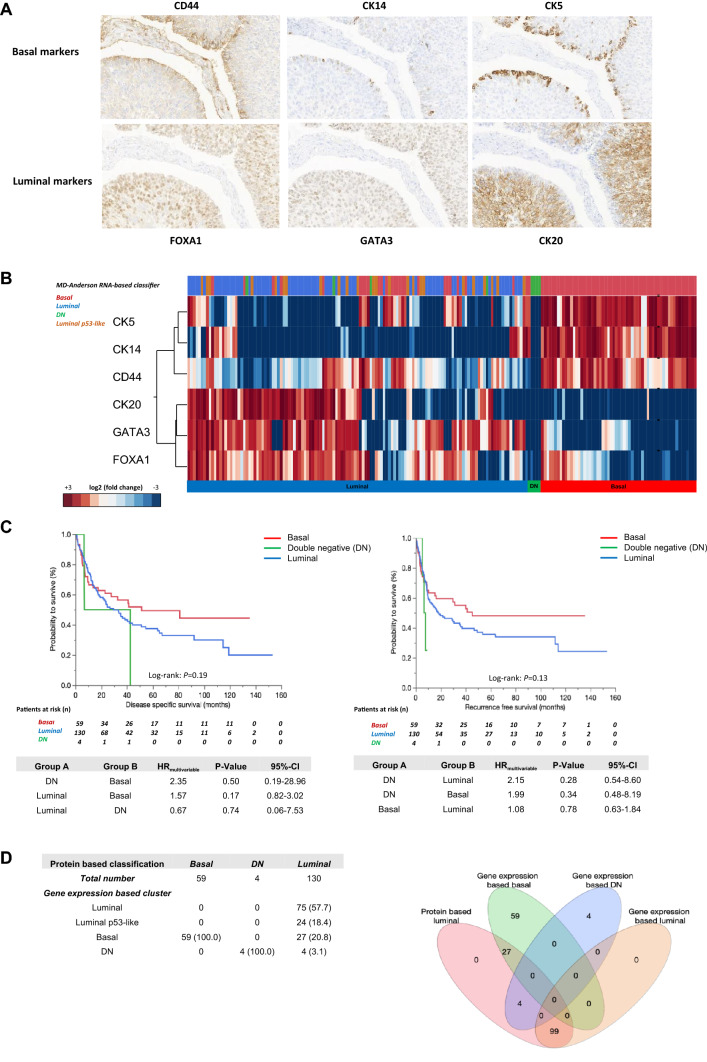
**A** Representative images of CD44, CK14, CK5, FOXA1, GATA3 and CK20 (magnification of all images 200 ×). **B** Unsupervised hierarchical clustering of these subtyping markers reveals three protein-based clusters: basal, luminal and double-negative (DN). **C** Univariable Kaplan–Meier regression of disease-specific survival (DSS) and recurrence-free survival (RFS) based on protein expression derived subtypes. Table shows total patients/phenotype with the following columns showing the number of patients at risk in 20-month increments. Multivariable Hazard risk (HR) ratios are shown. **D** Left panel: comparison of gene expression and protein-based subtypes. Right panel: a Venn diagram was constructed to highlight the distribution of discordant classifications of gene expression-based subtypes in the luminal protein cluster

Protein-based subtyping was carried out by applying a six-marker subtyping panel proposed by the BCMTG. Representative images of IHC stainings are shown in Fig. 2A. Unsupervised hierarchical clustering assigned 130 as luminal (67.4%), 59 tumors as basal (30.6%; Fig. 2B) and four tumors as DN (2.0%). Assignment to these protein subclasses did not correlate with survival (DSS: log-rank *P* = 0.19; RFS: log-rank *P* = 0.13; Fig. 2C). The 59 basal and four DN tumors were classified 100% concordantly with both approaches (mRNA vs. protein). 99 (76.2%) of 130 luminal classified tumors concordantly showed luminal mRNA subtypes, while 27 were classified as basal (20.8%) and 4 (3.1%) as DN by gene expression (Fig. 2D). To illustrate heterogenous distribution of mRNA subtypes within the luminal protein subtype group, a Venn diagram was constructed (Fig. 2D).

### Histological-based subtypes in MIBC and associations with mRNA and protein subtypes

**Fig. 3:**
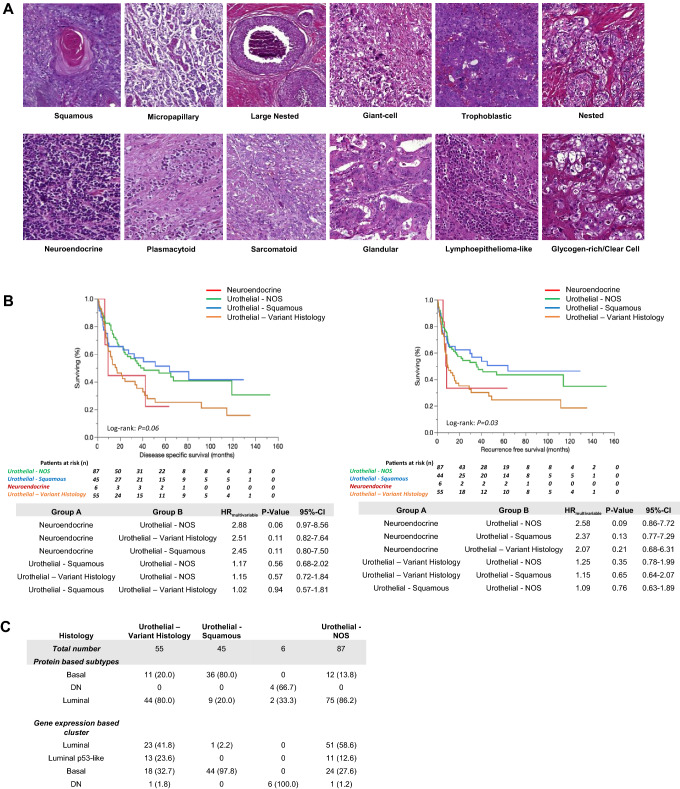
**A** Representative HE images of histological MIBC subtypes of the present MIBC cohort: squamous differentiation, micro-papillary, large nested, giant cell, trophoblastic, nested, neuroendocrine, plasmacytoid, sarcomatoid, glandular, lymphoepithelioma-like, and glycogen-rich/ clear cell. **B** Univariable Kaplan–Meier regressions of disease-specific (DSS) and recurrence-free survival (RFS) based on histological subtypes. Table shows total patients/phenotype with the following columns showing the number of patients at risk in 20-month increments. Multivariable Hazard risk (HR) ratios are shown. **C** Comparison of gene expression-based subtypes, protein-based subtypes and histological subtypes

87 tumors (87/193; 45.1%) presented with conventional morphology (not otherwise specified, NOS), 55 tumors (55/193; 28.5%) with variant histology, 45 tumors (45/193; 23.3%) with squamous differentiation and 6 tumors (6/193; 3.1%) with pure neuroendocrine differentiation. Representative images are displayed in Fig. 3A. Histological subtypes did not correlate with DSS (log-rank: *P* = 0.06), while a significant association could be found with RFS (log-rank: *P* = 0.03). However, in multivariable Cox proportional hazard regressions adjusted for clinico-pathological co-variables, no significant associations could be found for DSS or RFS (Fig. 3B). Figure 3C summarizes associations with gene- and protein-expression-based subtypes: NOS MIBC mainly showed luminal subtypes (gene expression subtyping: 62/87, 71.3%; protein subtyping: 75/87, 86.2%), squamous MIBC mainly basal subtypes (gene expression subtyping: 44/45, 97.8%; protein subtyping: 36/45, 80.0%), MIBC with variant histology mainly luminal subtypes (gene expression subtyping: 36/55, 65.5%; protein subtyping: 44/55, 80.0%), and pure neuroendocrine MIBC mainly DN subtypes (gene expression subtyping: 6/6, 100%; protein subtyping: 4/6, 66.7%). Compared to NOS MIBC, squamous tumors significantly associated with basal subtypes (P < 0.001) and neuroendocrine tumors with double-negative subtypes (*P* < 0.001).

## Discussion

Recent discovery of molecular subtypes in MIBC offers a possible and promising route to improvement of personalized treatment strategies for MIBC patients [23]. The present study was conducted to i) explore the prognostic impact of two different subtyping approaches (mRNA- and protein-based) in a consecutive population-based cystectomy cohort, (ii) to explore the clinical utility of a protein-based subtyping approach, and (iii) to explore associations of histological MIBC subtypes with molecular tumor subtypes.

Several studies suggested that molecular MIBC subtypes correlate with prognosis and response to neoadjuvant chemotherapy [10, 11]. However, recent meta-analysis of 2411 and 1750 inhomogeneously collected transcriptomic profiles of UBC were only able to show significant prognostic differences between MIBC with luminal-papillary-like expression profiles compared to neuroendocrine-like subtypes, which present with well-known aggressive clinical behavior [10, 24]. In addition, it has to be mentioned that stromal invasive carcinomas have frequently been included in these studies, and no systematic pathological re-review has been conducted in the underlying original patient cohorts. Therefore, these data might be biased by early-invasive papillary carcinomas not matching the criteria of MIBC, which have a significantly better baseline prognosis than true MIBC [24, 25]. In contrast to these meta-analyses and matching with this hypothesis of a systematic bias by early-invasive carcinomas, a study by Kollberg et al. failed to demonstrate a prognostic impact of molecular subtypes in a population-based consecutive cystectomy series with 519 patients who underwent radical cystectomy between 2006 and 2011 in Sweden [26]. Neither gene expression consensus subtypes nor protein-based subtyping showed any association with clinical outcome [26]. These observations are in line with our present findings in 193 consecutively collected MIBC patients where no association of mRNA- or protein-based subtyping with DSS or RFS could be found. These huge differences in terms of clinical outcomes between inhomogenously collected large meta-analysis cohorts and homogenous consecutive population-based cystectomy cohorts underlines the urgent need for prospective observational multicenter trials to investigate the true prognostic and predictive potential of molecular MIBC subtypes. Prospective validation of outcome as well as predictive effects by molecular subtypes will be critical to evaluate the utility of genomic subtyping.

Another major issue of molecular MIBC subtyping beside inconsistent data on the prognostic value is the mode of subtype assessment. Most subtyping approaches including the recently published consensus subtyping approach base on whole transcriptome mRNA profiling, which is a time-consuming and expensive method, which requires high RNA quality and huge bio-informatical resources [27]. Thus, the BCMTG defined an IHC marker panel with the intent to provide a reduced consensus panel of routinely applicable IHC markers, which should be sufficient to determine luminal and basal differentiation [22]. By applying this IHC marker panel, we were able to demonstrate a substantial overlap with gene expression-based subtypes (83.9%). However, discordant classifications occurred in 16.1% and were mainly observable in luminal tumors. Similar to our findings, Guo and coworkers’ evaluation of GATA3 and CK5/6 correctly identified approximately 80% of gene expression-based molecular subtypes among 74 MIBC cases. Based on these results, Guo et al. suggested to implement this two-marker panel as a simple immuno-histochemical classifier in routine diagnostic settings [27]. Our results indicate that the accuracy of predicting gene expression-based subtypes can be slightly increased by adding other subtype-specific markers; however, a group of discordantly classified tumors remains. Upcoming studies are necessary to explore if discordant classifications are clinically relevant. Identified subtypes among this study are more general and the consensus classification was not applied.

Conventional histopathological assessment is still the most important prognostic “marker” in MIBC [2]. Previous studies indicated a negative prognostic effect of variant histology in MIBC compared to NOS morphology [28]. In our present study, histological subtypes of MIBC were not independent predictors for DSS or RFS, which can be attributed in part to a smaller study cohort. Interestingly, histological subtypes of MIBC are strong indicators for specific mRNA and protein subtypes, e.g., squamous differentiation as indicator for basal subtypes, which has been indicated in previous studies [5, 14]. In contrast, NOS morphology and variant histology (especially micropapillary, nested and plasmacytoid morphology) strongly correlated with luminal phenotypes, which has only been indicated in small previously published case series [20, 21, 29].

Limitations of our study are the retrospective character and the relatively low number of analyzed patients. Due to the lack of NAC, we were not able to analyze the possible predictive value of these molecular subtypes, which could be of clinical relevance. The prevalence of variant histology in our cohort is higher than in previously reported cohorts. This might be caused by a lack of neoadjuvant treatment (NAC) with well-preserved residual tumors after TURB, by full embedding of the complete tumor mass according to standardized mapping methodologies applied in our institution [18, 19], and potentially due to a higher awareness of variant histology based on our primary research interest in bladder pathology.

## Conclusion

Matching with observations of the Lund group, we demonstrate that neither gene expression nor protein-based subtyping associates with patient outcomes in a population-based cystectomy cohort. Both subtyping strategies show substantial overlaps, thus indicating that protein-based subtyping could be used for MIBC subtyping in daily practice as a cost-efficient alternative to transcriptome sequencing. In addition, we provide evidence that histological subtypes correlate strongly with mRNA and protein molecular subtypes.

## Supplementary Information

Below is the link to the electronic supplementary material.

Supplementary file1 (XLSX 16 KB)
